# Loss to follow-up tuberculosis treatment and associated factors among adults attending at public health facilities in Warder District, Somali Regional State, Eastern Ethiopia

**DOI:** 10.3389/fpubh.2023.1151077

**Published:** 2023-05-10

**Authors:** Mohammed Birhane, Shambel Mekonnen, Tariku Dingeta, Zelalem Teklemariam

**Affiliations:** ^1^Galadi Woreda Health Office, Dollo Zone, Somali, Ethiopia; ^2^College of Health and Medical Sciences, School of Medical Laboratory Sciences, Haramaya University, Harar, Ethiopia; ^3^College of Health and Medical Sciences, School of Public Health, Haramaya University, Harar, Ethiopia

**Keywords:** tuberculosis, loss to follow-up, treatment outcome, Warder, Somali region, Ethiopia

## Abstract

**Background:**

Tuberculosis is a major public health problem worldwide, particularly in resource-limited countries. Loss of follow-up during treatment is one of the major obstacles in the fight against tuberculosis, which has serious implications for patients, their families, communities, and health service providers.

**Purpose:**

To assess the magnitude of the loss to follow-up tuberculosis treatment and associated factors among adults attending public health facilities in Warder District, Somali Regional State, eastern Ethiopia from November 02–17, 2021.

**Methods:**

A 5-year (from 1 January 2016 to 31 December 2020) retrospective study was conducted on 589 adult tuberculosis treatment records. Data were collected using a structured data extraction format. Data were analyzed using STATA version 14.0 statistical package. Variables with *P* < 0.05 in the multivariate logistic regression analysis were considered statistically significant.

**Results:**

A total of 98 (16.6%) TB patients failed to follow up with their treatment. Age between 55 and 64 years (AOR = 4.4, 95% CI: 1.9–9.9), being male (AOR = 1.8, 95% CI: 1.1–2.9), living more than 10 km away from a public health facility (AOR = 4.9, 95% CI:2.5–9.4), and having a history of tuberculosis treatment (AOR = 2.3, 95% CI: 1.2–4.4) were associated with a higher likelihood of not following up, while having a positive initial smear result (AOR = 0.48, 95% CI: 0.24–0.96) was associated with a lower probability of not following up.

**Conclusion:**

One out of six patients was lost to follow-up after initiating their tuberculosis treatment. Hence, improving the accessibility of public health facilities with a special focus on older adults, male patients, smear-negative patients, and retreatment cases is highly warranted among TB patients.

## Introduction

Tuberculosis (TB) is a communicable disease that affects the lungs (Pulmonary TB) as well as other organs of human body in cases of Extrapulmonary TB. Many countries have experienced rapid, sustained declines in tuberculosis deaths during the past 50 years (1). However, it is still among the top 10 causes of death worldwide. Globally, ~10 million people developed TB, and 1.4 million died in 2019. The World Health Organization (WHO) states that African and Southeastern Asian regions accounted for 85% of TB deaths in HIV-negative and HIV-positive people (2). Ethiopia is among the top 20 high-burden countries for TB, with at least 100,000 incident cases of TB in 2019 (3).

Tuberculosis can be treated, prevented, and cured. Approximately 85% of people who develop TB disease can be successfully treated with a 6-month drug regimen. Since 2000, TB treatment has saved more than 60 million lives. However, despite the efforts to achieve universal health coverage (UHC), millions of people still lack access to proper diagnosis and care (4).

The directly observed therapy short course (DOTS) was implemented in 1991 in Ethiopia. Despite the extensive expansion of DOTS services and the massive involvement of health extension workers (HEWs) in TB prevention and control activities, many TB patients are still failing to complete their treatment and achieve a cure (5, 6).

Loss to follow-up (LTFU) during treatment is one of the major obstacles in the fight against tuberculosis treatment and control. It presents serious implications not only for the patients themselves (including prolonged illness, the development of clinical complications, the development of drug resistance, and premature death), but it has the potential to lead to the spread and outbreaks of drug-resistant bacilli in their families, communities, and health service providers (7).

The proportion of LTFU varied considerably among different countries, different types of TB, and different patient populations. Research evidence reported in South Ethiopia indicated an 11.2% incidence of LTFU (8), 13.5% in Jimma, Ethiopia (5), 6.8% in China (9), 12.5% in Georgia (10), 18.1% in Brazil (11), and as high as 44.9% in Mozambique (12).

Several studies have identified various factors that contribute to patients failing to follow up on their TB treatment, including being male, advancing age, alcohol abuse among men, living in rural areas, moving within the country, distance from healthcare facilities, migration to another country, TB treatment, and weight loss (5, 9, 10, 13–16).

Knowledge of the magnitude of LTFU and its associated factors is essential for successful TB control and the optimal delivery of healthcare services in resource-poor settings (17). However, there are no published data on the proportion of loss to follow-up and its associated factors among patients enrolled in first-line tuberculosis treatment in the current study area. Therefore, this study assessed the magnitude of loss to follow-up tuberculosis treatment and associated factors among adults attending public health facilities in Warder District, Somali Regional State, and eastern Ethiopia. The results of this study could aid in planning measures that can reduce the percentage of patients who discontinue their treatment. The district and zonal health offices will also use the study's data as a baseline to revise or strengthen their tuberculosis treatment and control plans.

## Materials and methods

### Study area and period

This study was conducted at public health facilities in Warder District from 2 November to 17, 2021. Warder district is found in the Dollo zone (formerly known as Warder Zone), the eastern part of the Somali region. It is located about 1,152 km from Addis Ababa, the capital city of Ethiopia, and 528 km from Jigjiga, the capital city of the Somali region. Currently, Warder District has one primary hospital, two public/government health centers, and more than seven private clinics. Tuberculosis treatment and follow-up are provided at Warder primary hospital and two government health centers (Kurtunle and Inlaley). The health facilities had functional laboratories for TB sputum smear microscopy. The follow-up and care of patients with TB were provided based on the diagnosis and linkage of patients to DOTS in the TB clinic. Based on the national TB treatment guidelines, patients enrolled in first-line TB treatment receive a combination of drugs for 6–8 months.

### Study design and populations

A 5-year retrospective register-based cross-sectional study was conducted on 589 systematically selected adult patients enrolled in TB treatment at all public health facilities in Warder District from 1 January 2016 to 31 December 2020. This study included all adult TB patients who were registered in the TB unit log book with known treatment outcomes at public health facilities from 1 January 2016 to 31 December 2020. All adult patients whose treatment outcomes were not documented in the facility's tuberculosis treatment logbook and who were transferred out to other health facilities were excluded from the study.

### Sample size and sampling technique

The sample size was calculated with one slope regression Cox model by using Stata 14.0 Software power, considering an adjusted odds ratio (AOR) of 1.94 (by taking weight loss after starting TB treatment vs. no weight loss) and the probability of LTFU among adult patients enrolled in TB treatment as 0.135 from a study conducted in Jimma (5), 80% power, 95% CI, and a 10% proportion of withdrawal. The final sample size was 589.

**Figure 1:**
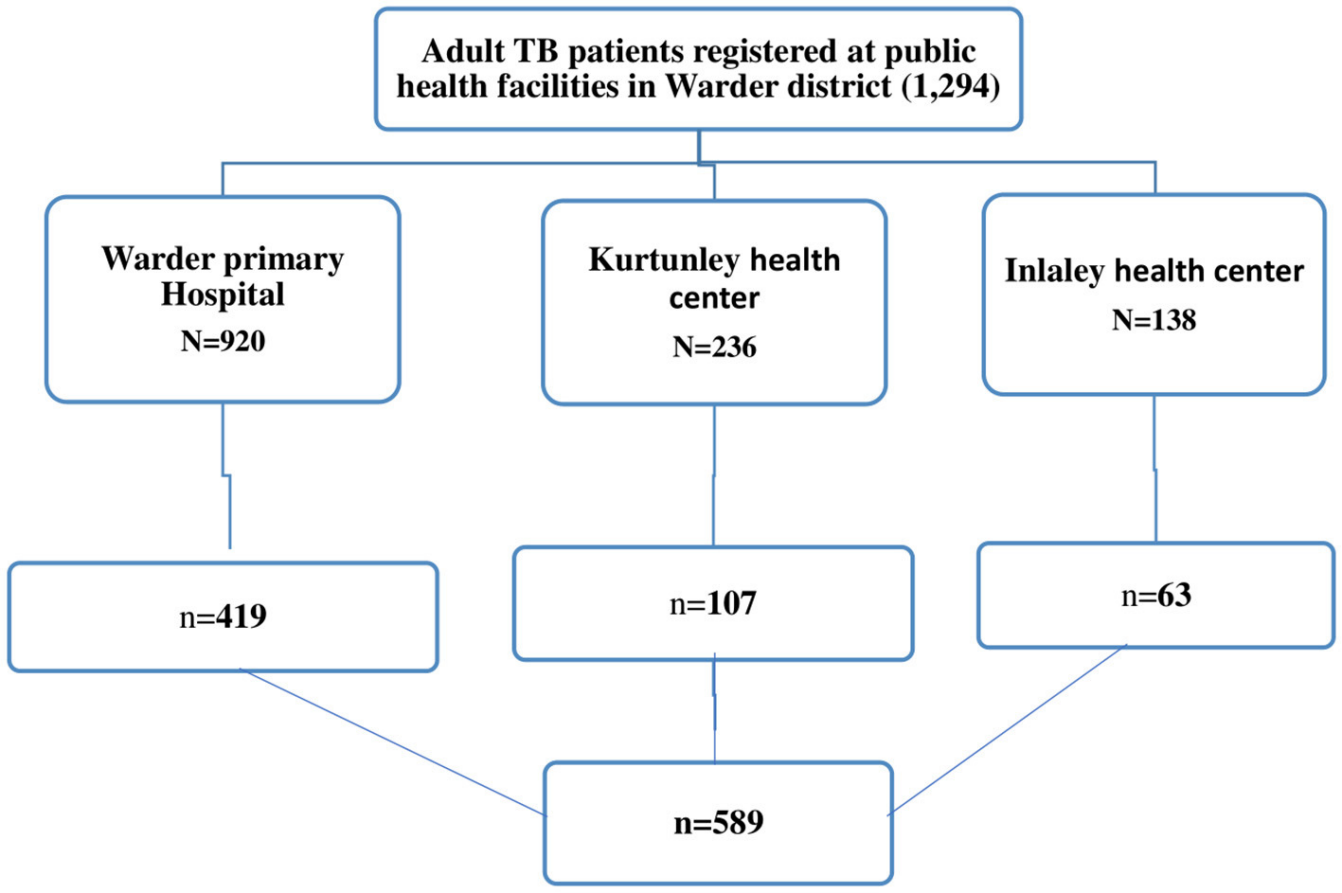
*N*, all adult patients enrolled in TB treatment during data retrieval period; *n*, sample size. Diagrammatic illustration of sampling procedure and sampling technique for LTFU and associated factors among adult Tuberculosis patients treated at public health facilities in Warder District, Somali region, East Ethiopia, 2021.

Warder district has three public health facilities (Warder primary hospital, Kurtunle, and Inlaley health center) that provide TB diagnosis, treatment, and follow-up services. The final sample size for the study was allocated proportionally to the total number of adult patients enrolled in tuberculosis treatment at each facility during the review period. study participants were selected from health facility TB register book through a systematic sampling technique until it reached the allocated sample size (Figure 1).

### Operational definitions

Weight loss after treatment initiation: Defined as a decrease in baseline weight of 2 kg within 4 weeks after the start of anti-TB drugs (18).

History of TB treatment: A patient was considered to have a history of TB treatment if, during admission, the patient was categorized under either retreatment or relapse in the health facility's TB register.

Type of TB: It refers to a type of tuberculosis that the patient has been diagnosed with and enrolled in treatment for, which is classified as Smear Positive Pulmonary TB (SPPTB), Smear Negative Pulmonary TB (SNPTB), and Extra Pulmonary Tuberculosis (EPTB).

### Data collection methods

Data collection was conducted by four healthcare workers (two BSc nurses and two health officers) using a structured data abstraction format prepared in English. The data abstraction format was developed from the WHO national treatment guidelines for TB treatment and other similar studies on the assessment of LTFU among TB patients in other areas (4, 5, 15), as well as consideration of known predictors that have an effect on LTFU in the area.

### Data quality control

About 2 days of training were given to data collectors and supervisors. The training mainly focused on extracting data from the TB unit register and patient records and filling out the data collection format while maintaining the confidentiality of information. The data collection tool was pretested before the actual data collection time. All the data abstraction formats were checked daily to ensure that they were appropriately filled. Double data entry was performed to ensure consistency in data entry. In addition, the quality of data collection was ensured through close supervision of the data collectors.

### Data processing and analysis

The data were entered and cleaned using Epi DATA version 3.02 and analyzed using STATA version 14.0 statistical package. Both descriptive and analytical methods were used for data analysis. The primary outcome of interest was the treatment outcome of LTFU, defined as a patient who took TB treatment for at least 1 month and discontinued treatment for more than eight consecutive weeks or more (19). Factors associated with LTFU were calculated using bivariate and multivariate logistic analysis. Variables with *P* ≤ 0.05 at a 95% confidence interval (CI) in multivariate analysis were considered statistically significant.

## Results

### Socio-demographic characteristics

In this study, a total of 589 adult patient records were reviewed. A total of 419 (71.1%), 107 (18.2%), and 63 (10.7%) TB patients' documents were reviewed from Warder Hospital, Kurtunley Health Center, and Inlaley Health Center, respectively. The study participants had ages ranging from 15 to 85, with a median age of 34 years (Inter Quartile Range (IQR): 26–47 years). About 180 (30.6%) of the participants were 25–34 years old. The majority of study participants were male (56.7%), the distance from their residence to the health facility was <10 km (56%), and they were urban residents (59.3%). About 22.8% of the study participants were selected from 2020 TB treatment-enrolled patients (Table 1).

**Table 1:** Socio-demographic characteristics of adult patients enrolled in tuberculosis treatment at public health facilities in Warder District, Somali Region, East Ethiopia 2021.

**Variable**	**Category**	**Number**	**Percent**
Age (years)	15–24	122	20.7%
	25–34	180	30.6%
	35–44	110	18.7%
	45–54	85	14.4%
	55–64	67	11.4%
	65 years and above	25	4.2%
Sex	Female	255	43.3%
	Male	334	56.7%
Distance of residence from health facility (Km)	<10	330	56%
≥10	259	44%
Residence	Urban	349	59.3%
	Rural	240	40.7%
TB risk group	Health care staff	13	2.2%
	Diabetes	32	5.4%
	Prisoner	21	3.6%
	Other congregated settings	523	88.8%
Year of enrollment for TB treatment	2016	97	16.5%
	2017	121	20.5%
	2018	108	18.3%
	2019	129	21.9%
	2020	134	22.8%

### Baseline clinical and treatment-related characteristics

The weight of the TB patients at enrollment ranged from 34.2 to 75.5 kg, with a mean weight of 56.2 kg and ± SD of ±8.1 kg. The majority of TB patients had Smear Positive Pulmonary TB (SPPTB; 47.2%), new (80%). About 2.7 % of TB patients were HIV seropositive, and the majority of them (93.7%) were enrolled in HIV care. A total of 503 (85.4%) of the study participants had no history of TB treatment. And start by among the 480 patients who completed the intensive phase and started the continuation phase, the majority of them (84.2%) had no weight loss from their pre-treatment weight (Table 2).

**Table 2:** Baseline clinical and treatment-related characteristics of adult patients enrolled in tuberculosis treatment at public health facilities in Warder District, Somali Region, East Ethiopia, 2021.

**Variable**	**Category**	**Number**	**Percent**
Type of TB	SPPTB	278	47.2%
	SNPTB	209	35.5%
	EPTB	102	17.3%
TB admission category	New	471	80.0%
	Relapse	27	4.6%
	Treatment after failure	14	2.4%
	Admission after LTFU	23	3.9%
	Transfer in	48	8.1%
	Other	6	1.0%
HIV sero status	Negative	420	71.3%
	Positive	16	2.7%
	Unknown	153	26.0%
Enrolled in HIV care	No	1	6.3%
	Yes	15	93.7%
Past TB treatment history	No	503	85.4%
	Yes	86	14.6%
Weight loss after initiation of TB treatment	No	404	84.2%
		Yes	76	15.8%

HIV, human immunodeficiency virus; SNPTB, smear negative pulmonary negative TB; SPPTB, smear positive pulmonary negative TB; EPTB, extrapulmonary TB.

### Treatment outcome in TB patients

**Figure 2:**
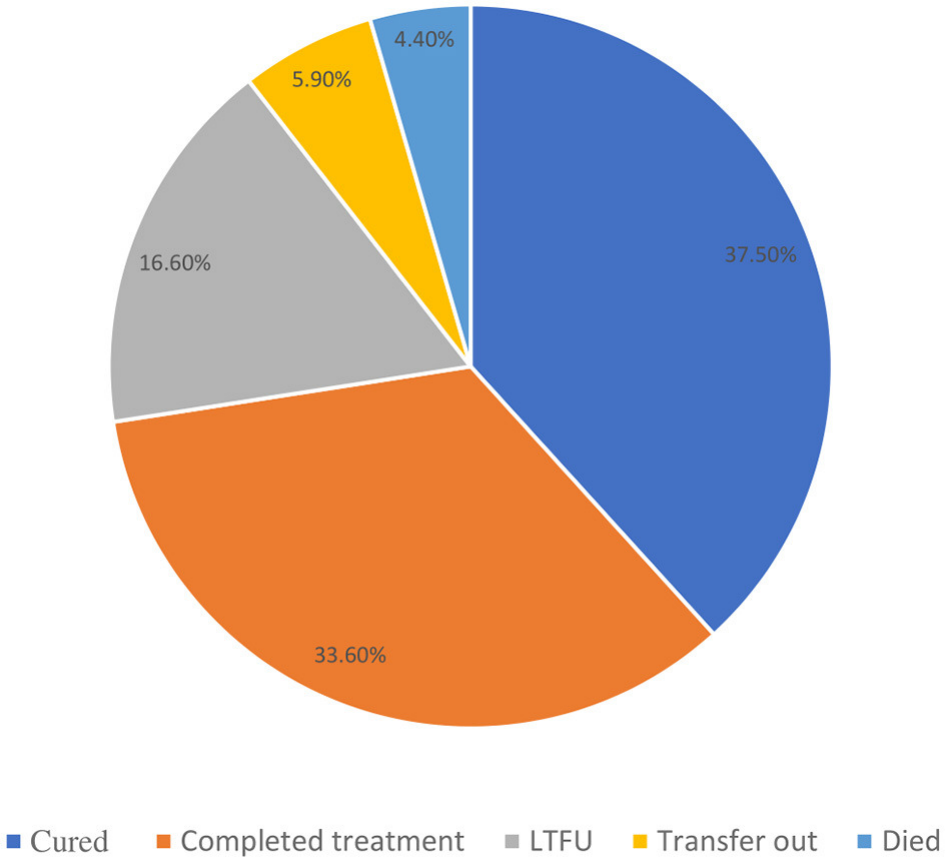
Treatment outcomes of adult patients enrolled in TB treatment at public health facilities in Warder District, Somali Region, East Ethiopia, 2021.

Out of 589 TB patients included in this study, 221 (37.5%) were cured, 198 (33.6%) completed treatment, 26 (4.4%) died, 9 (1.5%) failed treatment, 98 (16.6%) were LTFU (95% CI = 13.7–19.9%), 2 (0.3%) were moved to the DR TB register, and 35 (5.9%) were transferred to another health facility. The treatment success rate (the combination of those who were cured and treatment completed) among the study participants was 71.1% (Figure 2).

Out of 98 LTFUs, 64 (65.3%) were located at Warder Hospital, 27 (27.6%) at the Kurtunley Health Center, and 7 (7.1%) at the Inlaley Health Center. Among these LTFU patients, the majority (70.4%) were male, almost half (49.6%) were SNPTB patients, more than half (52%) were rural residents, and 70% had a residence ≥10 Km away from the public health facility at which they received TB treatment and were followed up. Sixty-two (63.3%) of those with LTFU were in the intensive phase, while 36 (36.7%) were in the continuation phase of TB treatment (Table 2).

### Factors associated with loss of follow-up

In the bivariate logistic regression analysis, variables such as age, sex, distance from the public health facility, residence, types of TB, and having a past TB treatment history with a *P*-value of <0.25 were candidates for the multivariate logistic regression analysis.

According to multivariate logistic regression analysis, ages 55–64 years, male sex, distance ≥10 km from the public health facility, types of TB, and having a history of TB treatment was found to be significantly associated with LTFU. Male TB patients were around two times more likely to discontinue TB treatment than female TB patients (AOR: 1.8; 95% CI = 1.1–2.9). TB patients aged 55–64 were four times more likely to fail to follow up on their TB treatment than those aged 15–24 (AOR: 4.2; 95% CI = 1.9–9.5). The odds of losing follow-up on treatment were five times higher among TB patients living more than 10 km away from public health facilities than those living within 10 km (AOR: 5.0; 95% CI = 2.6–9.7). Those with SNPTB (AOR: 2.3, 95% CI = 1.3–4.1) and EPTB (AOR: 2.2; 95% CI: 1.2–4.4) were two times more likely to fail to follow up on their TB treatment than smear-positive pulmonary TB (SPPTB) patients. Those with a history of TB were two times more likely to fail to follow up on their TB treatment than those with a history of TB (AOR: 2.2; 95% CI = 1.2–4.2; Table 3).

**Table 3:** Logistic regression analysis to identify factors associated with LTFU among patients enrolled in TB treatment at public health facilities in Warder District, Somali Region, East Ethiopia, 2021.

**Variable**	**Category**	**LTFU No. (%)**	**COR (95% CI)**	***P*-value**	**AOR (95% CI)**
Age in years	15–24	14 (11.5%)	1		1
	25–34	25 (13.9%)	1.2 (0.6–2.5)	0.54	1.4 (0.6–2.9)
	35–44	23 (20.9%)	2.0 (0.9–4.2)	0.05	2.1 (1–4.6)
	45–54	7 (8.2%)	0.7 (0.3–1.8)	0.45	0.7 (0.2–1.8)
	55–64	23 (34.3%)	4.0 (1.9–8.5)	0.00	4.2 (1.9–9.5)^*^
	65 and above	6 (24%)	2.4 (0.8–7.1)	0.10	2.1 (0.6–6.5)
Sex	Female	29 (11.4%)	1		1
	Male	69 (20.7%)	2.0	0.003	1.8 (1.1–2.9)^*^
Distance from health facility	<10 Km	29 (8.8%)	1		1
	≥10 Km	69 (26.6%)	3.8 (2.4–6.0)	0.00	5.0 (2.6–9.7)^*^
Area of residence	Urban	47 (13.5%)	1		1
	Rural	51 (21.3%)	1.7 (1.1–2.7)	0.01	0.5 (0.3–1.0)
Type of TB	SPPTB	30 (30.6%)	1		1
	SNPTB	46 (46.9%)	2.3 (1.4–3.8)	0.00	2.3 (1.3–4.1)^*^
	EPTB	22 (22.4%)	2.3 (1.2–4.2)	0.012	2.2 (1.2–4.4)^*^
Past TB treatment history	No	75 (14.9%)	1		1
		Yes	23 (26.7%)	2.1 (1.2–3.6)	0.00	2.2 (1.2–4.2)^*^

TB, tuberculosis; LTFU, loss to follow-up; Km, kilometer; CI, confidence interval; AOR, adjusted odds ratio; SNPTB, smear negative pulmonary negative TB; SPPTB, smear positive pulmonary negative TB; EPTB, extrapulmonary TB.

* P ≤ 0.05.

## Discussion

The prevalence of LTFU among TB patients on treatment in the study area was 16.6%. Being male, being in the ages of 55–64 years, living more than 10 km away from a public health facility, having a history of past TB treatment, and being diagnosed with smear-negative pulmonary negative TB or extrapulmonary TB were associated with loss to follow-up in adult TB patients.

This study showed that the overall prevalence of LTFU from TB treatment among study participants was 16.6%, However, it is higher compared to previous studies conducted in Jimma, Ethiopia (13.5%) (5), and Southern Ethiopia (11.2%) (8) Myanmar (9.1%) (16), China (6.8%) (9), and Georgia (12.5%) (10). The prevalence of LTFU in this study was lower compared to the LTFU rates of 17% in Norway (20), 29.6% in India (21), and 44.9% in Mozambique (12). This may be due to variations in study design, sample size, patient follow-up systems, study settings, sociodemographics, lifestyle, and healthcare access (in the current study, a predominantly pastoralist lifestyle is prevalent in the study area). This is consistent with the study report from Brazil (18.1%) (11).

Higher proportions of patient LTFU during the early periods or intensive phase of tuberculosis treatment pose a serious threat as it significantly contributes to drug resistance and transmission of TB infection to the close contacts of the patients who discontinued their treatment regimen (22). The majority of LTFU cases in this study (63.3%) occurred during the intensive phase of TB treatment. This finding is higher than 30.4% reported in study conducted in Jimma (5), this might be due to difference in tracking and monitoring of loss to follow up, accessibility of transportations, and health facilities.

This study has revealed that the incidence of LTFU is highest in patients aged 55–64 years. This finding is consistent with the results of a study conducted in China (9). The findings differ from a recent study on Haiti's national TB surveillance data (23), which indicated that the 25-34-year age group was associated with significantly higher LTFU rates than other age groups. This discrepancy might have stemmed from differences in social support systems, as the old age category of patients requires more social support from their communities in addition to the lengthy duration of treatment, which is long in terms of separation from patients' hometowns and daily routines.

This study has also shown that the male sex has a significant association with LTFU among TB patients. This finding is in line with a study conducted at Wolayta Sodo Referral Hospital, Southern Ethiopia (8) and Georgian national TB surveillance (10). The possible explanation for the male sex being associated with LTFU could have arisen from differences in men's and women's societal roles that men in the study area are usually tasked with looking after the livestock. Thus, men might not go to public health facilities to continue their treatment.

This study has also identified the distance of the patient's residence from the health facility as one of the factors significantly associated with LTFU. This is consistent with findings from a study conducted in Sheka Zone, Ethiopia (15). This can be justified because there might be a low urbanization rate, a lack of access, and/or a high transposition cost to go to treatment centers.

This study also found that TB patients with baseline smear-negative pulmonary and extrapulmonary TB had a significant association with LTFU. This finding is consistent with study conducted in South Africa (13). This might be due to difference of program attention given to this group of patients and the attitude of patients toward the proper diagnosis of their illness.

The previous history of tuberculosis treatment was also found to be significantly associated with LTFU in this study. This finding is consistent with studies conducted in Tajikistan (14) and South Africa (13), which have reported that patients who already received TB treatment were more likely to be LTFU from subsequent treatments, including patients with a relapse, former treatment failure, or treatment after LTFU. This might be explained by repeated experiences of adverse drug effects and suboptimal treatment, care, and follow-up services at health facilities.

### Strengths and limitations of the study

The main strength of this study is that it has assessed the magnitude of LTFU and associated factors among TB patients in a study area where there was previously no sufficient information on LTFU and challenges with regard to public transportation for TB patients and research activities. As part of the limitation of this study, the number of losses to follow-up might be overestimated or underestimated by the exclusion of patients with undocumented outcomes and undetermined treatment outcomes of transferred patients. In addition, the registration of TB patients did not incorporate reasons for treatment default due to the retrospective nature of the study.

## Conclusion

This study found a high prevalence of LTFU among TB patients in the Warder District. Age between 55 and 64 years, male sex, the distance of residence ≥ 10 km away from the public health facility, a diagnosis of smear negative pulmonary negative TB and extrapulmonary TB, and having a history of TB treatment history were found to be associated with LTFU among adult patients with TB treatment. Therefore, improving access to public health facilities and strengthening the Tuberculosis surveillance system with a special focus on older adults, males, smear-negative and EPTB, and patients with a history of TB. In addition, enhanced supervision and monitoring of DOTs strategy implementation, improved identification, and tracking of LTFU patients are warranted. Further, a prospective study on loss to follow up and associated factors among TB patients using a large sample size, a wide area, and reasons for loss to follow up is recommended.

## Data availability statement

The original contributions presented in the study are included in the article/Supplementary material, further inquiries can be directed to the corresponding author.

## Ethics statement

To conduct the study, permission was obtained from Haramaya University College of Health and Medical Science after an ethical review of the contents of the proposal by the university's Institutional Health Research Ethics Review Committee (IHRERC) Ref. No. IHRERC/177/2021, and a formal letter was written to the Warder District Health Office ensuring the approval of the proposal. Before starting the data collection, informed, voluntary, written, and signed consent was obtained from the head of the selected public health facilities for the study. This study did not extract information that identified the participants. Besides, data collectors used hand sanitizer and wore face masks to protect them from COVID-19. The patients/participants provided their written informed consent to participate in this study.

## Author contributions

MB participated in the conception, proposal development, data collection, writing up results, and drafting of manuscripts. SM, TD, and ZT participated in the proposal development, interpretation of the results, and critical reviewing of the manuscript. All authors read and approved the final version of the manuscript.
